# From alienation to deficitization: the subjectivity predicament of digital laborers

**DOI:** 10.3389/fpsyg.2026.1890203

**Published:** 2026-09-21

**Authors:** Zhaobin Chu, Qiuyu Fan

**Affiliations:** 1 Institute of Urban and Rural Economy, Anhui Academy of Social Science, Hefei, China; 2 Department of Philosophy, Shanghai Normal University, Shanghai, China; 3 Institute of Marxism, Anhui Academy of Social Sciences, Hefei, China

**Keywords:** alienation, digital labor, escape from freedom, Fromm, psychoanalytic social psychology, psycho-emotional deficitization, social character

## Abstract

Contemporary research on digital labor confronts a methodological tension: while mainstream occupational health psychology offers rich empirical evidence on the psychological distress of digital workers, it fails to capture the more fundamental predicament of subjectivity underlying this distress—namely, Why, When formal autonomy has been elevated to an unprecedented height, does workers’ inner experience become increasingly passive? We argue that this explanatory gap stems from implicit theoretical assumptions in mainstream psychology, which treats psychological needs as ahistorical, universal constants and presupposes the individual as a rational actor who calculates gains and losses in response to external pressures and resources. Fromm’ s psychoanalytic social psychology provides an alternative theoretical paradigm. Through the core concept of “social character,” Fromm reveals how socioeconomic structures operate on individuals not as external variables, but by shaping their deepest need structures and affective orientations, so that individuals “voluntarily” come to feel, think, and act in the manner required by the system. This article systematically elaborates the epistemological implications and methodological value of three core Frommian concepts—alienation, social character, and escape from freedom—and proposes that contemporary digital labor cultivates a “digital marketing character”: in the process of continuous self-curation, self-quantification, and self-commodification, workers experience alienation as self-realization. The psychological effect of this character structure is a diffuse state of “psycho-emotional deficitization”—a deep-seated experience of constantly feeling insufficiently productive and valuable regardless of objective performance. We critically examine Fromm’s own framework, acknowledging its limitations regarding universalist assumptions, normative presuppositions, and historical boundedness. We also situate this intervention within the broader landscape of critical traditions—including Foucauldian, psychosocial, psychodynamic, posthumanist, and platform capitalism scholarship—and explicitly delimit the contribution as a strategic bridge between critical theory and empirical work psychology. To move beyond theoretical speculation, we provide a systematic methodological roadmap with explicit, testable hypotheses, scale development strategies, and multiple research designs spanning qualitative, psychometric, longitudinal, SEM, mixed-methods, and comparative approaches. The contribution of this article is twofold: it furnishes psychology with a set of historically informed and incisive conceptual tools for understanding the subjectivity predicament in digital labor, and it builds a theoretical bridge between mainstream psychology and psychoanalytic social psychology.

## 1 Introduction: when autonomy becomes a burden—a psychological problem requiring a new paradigm

Contemporary digital labor presents a phenomenon that bewilders mainstream psychology: workers have acquired unprecedented formal autonomy—the freedom to decide when, where, how intensely, and with whom to work—yet their inner experience is often characterized by powerlessness, anxiety, and diffuse self-doubt. One video creator with hundreds of thousands of followers described her state in an interview: “Every time I post a piece of work, I only allow myself 5 min to celebrate the data, whether good or bad. After those 5 min, I have to start thinking about the next topic.”

These accounts point to a particular form of psychological distress: it breeds beneath the veneer of “autonomous choice,” and its nature is not a direct reaction to work intensity, but a deeper, diffuse unease about self-worth. Mainstream occupational health psychology has extensively studied such distress, including burnout, anxiety, depression, and work addiction (Matilla-Santander et al., 2025), yet a fundamental question remains insufficiently interrogated: Why, When formal autonomy is maximized, does workers’ subjective experience tend toward passivity and emptiness?

### 1.1 The explanatory logic of mainstream psychological frameworks and its limits

To grasp the theoretical significance of this question, it is necessary to first examine the explanatory logic of mainstream psychological frameworks. The Job Demands-Resources (JD-R) model understands the psychological distress of digital workers as an imbalance between job demands and job resources: algorithmic management generates persistent pressure, while the lack of social support undermines coping resources, and together they lead to burnout (Bakker and Demerouti, 2017; Demerouti et al., 2001; Schaufeli and Bakker, 2004). The Effort-Reward Imbalance (ERI) model emphasizes that workers invest substantial time and emotional energy, but the platform’s algorithmic pricing and evaluation mechanisms render rewards highly uncertain, and this imbalance triggers sustained stress responses (Siegrist, 1996). Self-determination theory (SDT) approaches the issue from the perspective of basic psychological needs, suggesting that the needs for autonomy, competence, and relatedness may all be simultaneously frustrated in digital labor (Bartholomew et al., 2011; Ryan and Deci, 2000; Sheldon et al., 2001) systematically analyzed how algorithmic control replaces traditional managerial authority as the new terrain of labor process contestation; Parent-Rocheleau and Parker (2022) further delineated the multiple mechanisms through which algorithmic management affects workers.

These frameworks have amassed a wealth of valuable empirical knowledge, but their explanations encounter a systematic boundary: they all presuppose the individual as a rational actor who calculates gains and losses when facing external variables, yet they fail to explain the diffuse suffering that seems completely internalized and almost unconscious (Lindebaum and Langer, 2024). Concretely, this limitation manifests at three levels:

First, a weakness in explaining the “autonomy paradox.” The JD-R model treats autonomy as an inherently positive job resource (Schaufeli and Bakker, 2004), but in digital labor, heightened formal autonomy not only fails to yield the expected increase in wellbeing but may actually exacerbate psychological distress. Empirical research shows that the relationship between perceived “job autonomy” and workplace wellbeing among gig workers is mediated by work alienation, and that perceived algorithmic control moderates this relationship (Wan et al., 2024). This implies that “autonomy” in platform labor may not be genuine self-determination, but rather a “pseudo-autonomy” pre-framed by a specific social structure. When workers internalize this pseudo-autonomy as “my own choice,” external control is transformed into self-control, while their originally frustrated psychological needs can no longer be recognized by themselves as frustrated.

Second, an ahistorical presupposition about needs themselves. Self-determination theory treats autonomy, competence, and relatedness as cross-culturally universal psychological needs, but it brackets a deeper question: How are the concrete forms and modes of satisfaction of these needs shaped by social structures? A feudal peasant and a digital worker both “need” identity, but the former seeks it within a stable hierarchical order, while the latter searches for it within a fluid, quantified ranking system. The essence of this difference is that the social form of needs has undergone fundamental change, rather than merely a difference in the “degree of satisfaction.” A Frommian line of questioning thus enters: under a specific social structure, What has the individual “learned to need”? How has the logic of capital valorization transformed itself into the individual’s inner need structure?

Third, a lack of analysis of the social-structural roots of psychological distress. Mainstream frameworks treat stressors and resources as variables that can be externally observed and measured, with the individual’s psychological state being a function of the interaction of these variables (Maslach et al., 2001). Following this logic, if a correlation is found between “algorithmic management” and “burnout,” policy recommendations tend to settle on optimizing the algorithm or increasing resources. But these frameworks cannot interrogate why, at a particular historical stage, a specific form of labor organization systematically pushes workers toward this specific form of distress, or whether the very “shape” of this distress carries some sort of social-structural information. The systematic review by Matilla-Santander et al. (2025) also notes that existing evidence primarily focuses on surface indicators of mental health, with insufficient attention to how platform labor profoundly shapes workers’ self-experience.

It is precisely these three explanatory boundaries that constitute the theoretical starting point of this article. To cross these boundaries requires the introduction of a different theoretical paradigm—an analytical tool capable of anchoring psychological phenomena within social structures, without dissolving subjectivity into a mere epiphenomenon of structure.

### 1.2 The paradigmatic value of Fromm’s psychoanalytic social psychology

Fromm’s psychoanalytic social psychology offers precisely such a paradigm. In disciplinary history, Fromm occupies a unique position: he received rigorous psychoanalytic training (studying within the Freudian tradition), was simultaneously deeply influenced by Marx’s historical materialism, and had profound academic affinities with the critical theory of the Frankfurt School (McLaughlin, 1998). This confluence of multiple intellectual traditions makes his theory different both from mainstream psychology’s tendency to abstract the individual from their socio-historical context and from certain radical social theories that reduce the individual entirely to a product of structure. O’Donnokoé et al. (2025), in a recent editorial for a special issue on Fromm, also emphasize that Fromm’s humanistic psychoanalysis still provides a valuable normative orientation in the face of contemporary social and political challenges.

The core value of the Frommian paradigm lies in a key methodological mediating concept—“social character.” Social character refers to the nucleus of the character structure shared by the majority of members of a specific group; its function is “to channel the psychic energy of the members of a given society in such a way that their behavior is not a matter of conscious decision whether or not to follow the social pattern, but a matter of wanting to act as they have to act” (Fromm, 1947, p. 283). The epistemological significance of this concept is that it breaks the dichotomy between “individual psychology” and “social structure” prevalent in mainstream psychology. Social character is neither purely personal, distinct from personality traits in the sense of individual differences; nor is it purely social, different from “norms” or “institutions” external to the individual. It is the interface between society and the individual—social structure enters the individual’s deepest needs, emotions, and desires through the shaping of social character, and the individual, by internalizing this social character, “voluntarily” acts in ways that adapt to the requirements of the social system.

Complementing this concept are Fromm’s theories of alienation and escape from freedom. Fromm extends alienation from the economic sphere into a psychological dimension, making it a mode of “psychic experience”—the person does not experience himself as the source of his own power and as an active agent, but rather experiences himself and others as objects to be exchanged and quantified (Fromm, 1955). Escape from freedom reveals a paradoxical psychodynamic: after modern individuals have acquired unprecedented formal freedom, they develop psychological mechanisms for escaping it precisely because they lack sufficient inner strength to bear the aloneness and responsibility that such freedom entails (Fromm, 1941).

These three concepts constitute a complete analytical apparatus: social character connects structure and individual, alienation describes the experiential quality of this connection, and escape from freedom explains why the individual remains within this experience. It is this apparatus that can potentially provide insights, unavailable in mainstream psychology, to answer the paradox of the “passivization of subjectivity” among digital workers.

One might question whether concepts developed in the context of mid-twentieth-century industrial capitalism remain suitable for understanding the substantially different historical configuration of contemporary platform-mediated labor. The historical distance is real, yet it does not invalidate Fromm’s framework. Fromm’s core concepts—social character as a mechanism for channeling psychic energy into socially required forms, alienation as a mode of psychic experience, escape from freedom as a psychodynamic principle—are formal architectures whose empirical content is historically variable. The “digital marketing character” we propose is not a simple application of the marketing orientation to a new setting but a deliberate reformulation reflecting the qualitatively new conditions of platform-mediated labor: compulsory self-curation, internalized self-quantification, and self-commodification experienced as autonomy. We are not projecting contemporary problems onto Fromm; we are drawing on his formal architecture to generate new, empirically testable concepts adequate to present conditions.

### 1.3 The theoretical task and structure of this article

Based on the above problematization, this article aims to accomplish the following theoretical tasks: drawing on Fromm’s concepts of social character, alienation, and escape from freedom, it will construct a theoretical framework to explain the specific form of psychological distress characteristic of digital workers, and from this framework, derive theoretical propositions amenable to empirical testing.

On a more general level, this article also represents a contributory argument concerning psychological methodology. We seek to demonstrate how psychoanalytic social psychology can offer psychology a set of conceptual tools with unique explanatory power and methodological operability for understanding the problem of subjectivity in contemporary forms of work. The positioning of this article is to provide a complementary paradigmatic perspective at the boundary where mainstream frameworks reach their explanatory limits, rather than a negation of those frameworks.

The structure of this article is as follows: first, we review the theoretical roots of Fromm’s core concepts, focusing on the methodological implications of social character, alienation, and escape from freedom, followed by a critical examination of the limitations of his framework; second, we map out the dialogical space between the Frommian paradigm and existing psychological research on digital labor, supplemented by engagement with the broader interdisciplinary literature; next, we move into the core of theoretical construction, systematically elaborating the causal nexus among digital marketing character, psycho-emotional deficitization, and escape from freedom; then we provide a systematic methodological roadmap with explicit, testable hypotheses and multiple research designs; and finally, we discuss the theoretical contributions of this paper.

## 2 The epistemological foundations of Fromm’s psychoanalytic social psychology

Before entering the concrete analysis of digital labor, it is necessary to systematically elaborate the theoretical roots of Fromm’s core concepts, aiming to clarify what kind of understanding and explanatory mode these concepts offer to psychology, what their epistemological presuppositions are, what they allow us to see that mainstream frameworks cannot, and where their methodological operational space lies.

### 2.1 From Freud to Fromm: the social turn in psychoanalysis

To understand the paradigmatic value of Fromm, one must first understand the critical turn he made in the history of psychoanalytic theory. Freudian psychoanalysis traced the deep driving forces of human behavior to biological instincts—libido and the aggressive instinct—with society establishing civilizational order through the repression and sublimation of these instincts. In this framework, society stands as a repressive force opposite the individual, and psychological conflict is the eternal war between instinct and civilization.

Fromm made a fundamental revision to this framework. He argued that the key feature distinguishing humans from other animals lies precisely in the weakening of instinctual determinism: humans are no longer governed by fixed instinctual patterns, thereby acquiring a certain “freedom,” but at the same time, they lose the natural connectedness to the world that instincts provide (Fromm, 1941, 1947). This “existential dichotomy” generates uniquely human psychological needs—what Fromm called the needs for relatedness, transcendence, rootedness, a sense of identity, and a frame of orientation. Although these needs are rooted in the existential condition of humans, their concrete modes of expression and satisfaction are shaped by specific socioeconomic structures.

The theoretical consequences of this revision are far-reaching. It means that psychoanalysis is no longer a science of eternal instincts, but is transformed into an analytical tool for examining the character structure and psychological experience of specific groups under specific historical conditions (Funk, 1998). Fromm’s view is that the “hoarding tendency” or the “marketing orientation” in capitalist society are not variants of a universal human psychological structure, but products of the mode of channeling human energy required for the functioning of a particular socioeconomic system. A society that needs enforced saving to accumulate capital must cultivate tendencies toward thrift and self-discipline; a society that needs continuously to create new demands to expand consumption must cultivate a desire structure of perpetual insatiability. Character structure is the anchoring device of social structure in the individual psyche (Fromm, 1947).

### 2.2 Social character: the methodological mediator between individual and society

The concept of “social character” is the methodological core of Fromm’s entire theoretical system (Riesman, 1950). If one were to summarize its epistemological function with a concise formula, it would be: socioeconomic structure → social character → individual behavior and experience. The arrows in this formula do not indicate a causal relationship—that would be too crude—but rather a relationship of structural coupling. Social structure does not “cause” social character, but rather “demands” and “selectively reinforces” certain character tendencies, making individuals with those tendencies more likely to “function normally” and find satisfaction within that social system.

The uniqueness of this concept lies in its transcendence of two common presuppositions in mainstream psychology. One may be called methodological individualism: treating the individual as the basic unit of analysis, with society being merely the result of the aggregation or interaction of individual actions. The other may be called environmental determinism: treating social structures (such as class position, working conditions) as external variables that act directly on the individual, who responds to these variables through processes such as “cognitive appraisal” or “social learning.” Fromm’s perspective differs from both: social character is internalized structure—it is the mode of existence of structure within the individual, yet it is also distinct from individual-level trait differences or cognitive responses to the environment.

In terms of understanding digital labor, this means: when discussing the psychological distress of digital workers, we are not merely discussing how the external stressor of “algorithmic management” leads to burnout through cognitive appraisal; we are also discussing a particular historical shaping within the workers’ deep need structure—they “need” to be continuously seen, rated, and recognized as “valuable individuals,” and this very need, in turn, supports the continuous operation of the platform economy. This is a deep psychological fact: structurally produced, yet simultaneously experienced authentically by the subject as a spontaneous need, which is far from a simple “false consciousness” narrative.

### 2.3 The psychological dimension of alienation: from Marx to Fromm

Fromm’s theory of alienation is one of his most original contributions within the Marxist tradition. In *Economic and Philosophic Manuscripts of 1844*, Marx located alienation primarily in the labor process—the worker is alienated from the product of labor, the activity of labor, species-being, and other human beings. Fromm (1955) expanded alienation from a category of political economy into a category of psychological experience that permeates the entirety of life.

As Fromm (1955) defined it, “By alienation is meant a mode of experience in which the person experiences himself as an alien. He has become, one might say, estranged from himself. He does not experience himself as the center of his world, as the creator of his own acts—but his acts and their consequences have become his masters, whom he obeys, or whom he may even worship” (p. 120). This experience can occur in labor, but also in consumption, leisure, and interpersonal relations. When a person buys a commodity in order to “have” it as a display of identity, they are alienated in consumption. When a person’s leisure activities are pursued not out of intrinsic interest, but because these activities are market-validated lifestyles that can be “shown off,” they are alienated in leisure. When a person views others as potential business opportunities with a certain “exchange value,” rather than as whole beings, they are alienated in interpersonal relations.

The methodological significance of this extension is that alienation is no longer a class-specific problem occurring only on the factory floor, but a diffuse psychological experience that can permeate all spheres of life under a particular social structure. This makes Fromm’s theory of alienation intrinsically suited for analyzing digital labor—because in digital labor, the boundaries between labor, consumption, leisure, and sociality have already dissolved (Fuchs, 2014; Terranova, 2000). A self-media creator films content during a gathering with friends: is this labor or leisure? Socializing or producing? When all domains of life are swept into the torrent of self-commodification, alienation is no longer an attribute of a specific activity, but becomes the backdrop of the entire lifeworld.

### 2.4 Escape from freedom: the psychodynamic masked by pseudo-autonomy

*Escape from Freedom* is Fromm’s most influential work, yet its core insights have been used very little in contemporary psychological research. The book reveals a fundamental paradox: after modern people acquired unprecedented freedom, they paradoxically developed psychological mechanisms for escaping it. The reason lies in the dual nature of freedom—it is both “negative freedom,” liberation from traditional bonds (freedom from), and “positive freedom,” the need to face the world’s uncertainties alone, make choices, and bear the consequences (freedom to). When individuals lack sufficient inner strength and external resources to bear the autonomy required by positive freedom, freedom becomes a burden, inducing feelings of isolation and powerlessness, which in turn activate the psychodynamic of escape.

Fromm analyzed three primary mechanisms of escape: authoritarianism (dissolving oneself into a powerful external force to gain security), destructiveness (eliminating one’s own powerlessness by destroying external objects), and automaton conformity (abandoning one’s individual self to become the kind of person the cultural pattern expects). The common essence of these three mechanisms is that the individual trades away an independent self in exchange for protection from the anxiety of aloneness.

This concept has direct inspirational value for understanding the pseudo-autonomy paradox in digital labor. If, as Fromm revealed, freedom can become a burden under specific conditions, then the celebrated “autonomy” in digital labor—the freedom to choose working hours, location, and intensity—may be not only a resource but also a source of pressure. When workers are granted high formal autonomy but lack genuine internal resources (such as a stable sense of self-worth) and external conditions (such as social security, collective bargaining power) to sustain this autonomy, they can easily develop the psychodynamic of escape from freedom: not fleeing the platform, but integrating themselves ever more actively into the platform’s logic, gaining a substitute for security by becoming the “most model worker.”

### 2.5 The limitations of Fromm’s framework and a qualified use

The preceding analyses of alienation, social character, and escape from freedom provide a powerful lens for understanding the psychodynamics of digital labor, yet no theoretical framework is without its historical and normative blind spots. We now turn to a critical examination of Fromm’s own theoretical framework. His postulation of universal human needs rooted in the existential condition has been critiqued by social constructionist and cultural psychological traditions, which argue that needs are constituted through historically specific discourses and practices rather than given by an ahistorical human essence (Burr, 2015; Gergen, 1985, 1991; Moscovici, 2000). His concepts of health and the productive orientation carry normative assumptions about human fulfillment that reflect specific cultural and historical values rather than universally valid standards (Foucault, 1965; Szasz, 1960). His conception of an authentic self that can be realized under the right social conditions has been challenged by poststructuralist and Lacanian perspectives, which question the very notion of a pre-given self to which one can be true (Foucault, 1988; Stavrakakis, 2007; Žižek, 1989). Moreover, translating his clinically grounded concepts into empirically operationalizable constructs inevitably involves a degree of phenomenological reduction—the rich, existential texture of his descriptions cannot be fully captured by psychometric scales (Westen, 1998).

In light of these limitations, this article adopts a qualified and pragmatic use of Fromm. His concepts are deployed as descriptive and explanatory tools rather than as normative prescriptions or metaphysical commitments. The “productive orientation” is treated not as an ideal workers are failing to achieve but as a construct whose buffering role against the deficitization–escape cycle is an empirical question to be investigated, not assumed. We do not offer Fromm’s framework as immune to critique; rather, we argue that, when used with appropriate critical caution, it provides a set of analytical resources that merit a place within the broader interdisciplinary conversation. The measure of their value will be their capacity to generate fruitful empirical research and illuminate dimensions of contemporary experience that other frameworks leave in shadow.

## 3 Psychoanalytic social psychology and digital labor studies: spaces of convergence

Before unfolding the theoretical construction of this article, it is necessary to clearly map out the “dialogical space” between the Frommian paradigm and existing psychological research on digital labor, supplemented by engagement with the broader interdisciplinary literature on platform capitalism, algorithmic surveillance, and digital subjectivity.

### 3.1 Existing achievements and unresolved questions in the psychological study of digital labor

Over the past decade, psychological research on digital labor has amassed substantial findings. The levels of burnout, anxiety, and depression among gig economy workers have been repeatedly measured, and factors such as algorithmic management, lack of social support, and job insecurity have been identified as significant predictors (Matilla-Santander et al., 2025; Vallas and Schor, 2020; Wood et al., 2019). Some studies have begun to attend to deeper mediating and moderating mechanisms, such as the mediating role of work alienation between autonomy and wellbeing (Wan et al., 2024), and how gamification mechanisms drive behavioral addiction. Lang et al. (2023) revealed the significant impact of perceived algorithmic control on the psychological exhaustion of gig workers. Gandini (2019) analyzed new forms of managerial control in the gig economy. van Doorn (2017) uncovered the intersecting dimensions of gendered and racialized exploitation in platform labor.

However, a few studies have touched upon the core issue that concerns this article but failed to offer an adequate explanation within existing theoretical frameworks. For instance, Wan et al. (2024) demonstrated that gig workers experienced more severe self-exploitation under conditions of high autonomy, a phenomenon that the JD-R model cannot fully explain. Similarly, Lang et al. (2023) found that perceived algorithmic control significantly intensified workers’ burnout, and that this burnout in turn mediated the relationship between algorithmic control and work engagement—suggesting that algorithmic management does not simply act as an external stressor, but intervenes more deeply in workers’ affective and motivational systems.

These “unresolved questions” point in the same direction: the explanation of certain core psychological experiences in digital labor requires a theoretical perspective capable of connecting the deep psychodynamics of the individual with the production requirements of the social structure.

### 3.2 Resonances of the Frommian tradition in contemporary organization studies

In recent years, critical organization studies have witnessed a clear scholarly turn back to Fromm’s theoretical tradition. Lindebaum and Langer (2024) proposed the concept of “psycho-emotional deficitization” in *Organization*, directly borrowing from Fromm’s theory of social character. They argue that workers under cognitive capitalism internalize a socially set ideal image of the “sufficiently good worker” and continuously compare their own present state against this ideal, thereby experiencing a diffuse feeling of “never being enough.” Fuchs (2020) explored how Fromm’s thought on communication can provide a critical perspective for understanding digital capitalism and algorithmic society. Moore (2018) systematically examined how digital technologies place workers under the pressure of self-quantification. Krüger and Johanssen (2014), using depth hermeneutics, analyzed alienation and unconscious dimensions in digital labor. These existing studies constitute the dialogical basis of this article, but they have yet to integrate Fromm’s concepts into a coherent analytical framework with implications for psychological theory and methodology.

### 3.3 Engaging the broader interdisciplinary literature

Beyond the psychological and organizational literature, the phenomena we address have been extensively examined across several key interdisciplinary streams. Platform capitalism studies establish the structural foundations: platforms extract data while externalizing risk onto workers (Srnicek, 2017; van Dijck et al., 2018; Woodcock, 2020). The governmentality and neoliberal subjectivity tradition, from Foucault, Foucault’s (1988, 2008) technologies of the self to Han’s (2017) psychopolitics and Bröckling’s (2016) entrepreneurial self, dissects how power operates through the constitution of self-governing, performance-oriented subjects. Complementing these, critical work on algorithmic management and the quantified self clarifies the technological infrastructure of internalized self-quantification (Pasquale, 2015; Zuboff, 2019; Lupton, 2016; Cheney-Lippold, 2017). Why self-commodification comes to feel autonomous is further illuminated by theories of emotional and free labor (Hochschild, 1983; Terranova, 2000; Jarrett, 2016) and empirically corroborated by ethnographies of platform work and “über-alienation” (Rosenblat, 2018; Glavin et al., 2021). Posthumanist approaches (Barad, 2007; Braidotti, 2013; Latour, 2005) challenge the anthropocentric assumptions of our framework, a limitation we recognize while maintaining that the first-personal phenomenology of suffering remains irreducibly psychodynamic.

Collectively, these bodies of work have substantially advanced our understanding of the structural, discursive, and technological conditions of digital labor. Yet they leave open a specific gap. The Foucauldian and psychopolitical traditions excel at tracing the normative techniques through which the “entrepreneurial self” is constituted, but they do not systematically theorize the affectively textured inner experience that results—the anxiety, deficiency, and passivity that suffuse workers’ self-experience. Sociological and platform capitalism studies illuminate external control mechanisms but stop short of explaining why workers so often internalize these mechanisms as constitutive of their own identity and self-worth. What is missing is a framework that connects the macro-level logics of platform capitalism to the deep psychological structure of the subject—a framework capable of showing how these conditions take up residence in workers’ needs, desires, and character structures. It is precisely this gap that a Frommian psychoanalytic social psychology is equipped to address.

### 3.4 Situating the contribution: a strategic bridge, not a theoretical void

We engage this broader critical field not to claim that Fromm fills a void no other tradition has addressed, but to make explicit the distinctive role a Frommian framework can play: a strategic bridge between existing critical traditions and empirical work psychology. Critical social psychology, the Foucauldian tradition, neoliberal subjectivity studies, the psychodynamics of work, and platform capitalism scholarship have all delivered powerful critiques of mainstream assumptions (Gergen, 1985; Foucault, 1988; Rose, 1999; Dardot and Laval, 2013; Hollway and Jefferson, 2013; Dejours, 2015; Srnicek, 2017). Our intervention does not compete with them; it strategically supplements them with a conceptual resource conspicuously absent from the methodological toolkit of mainstream work psychology—a psychoanalytic theory of social character that combines clinical depth with empirical tractability.

Fromm’s framework is suited to this bridging function because of its unique epistemological position: it is grounded in a clinical tradition attentive to subjective depth, yet it formulates its core concepts at a level of abstraction that can be translated into empirically operationalizable constructs without requiring prior acceptance of the full Lacanian or Foucauldian apparatus. Two complementary relationships are especially salient. First, Fromm and Foucault operate at complementary levels. Foucault provides the anatomy of techniques and rationalities through which self-governing subjects are constituted, while Fromm provides the phenomenology of the resulting inner experience—the anxiety, deficit, and flight from freedom. The social character concept specifies the psychological mechanism through which technologies of the self-become anchored in personality. Second, where platform capitalism scholarship explains structural conditions and psychopolitical theory offers philosophical diagnosis, we provide the characterological and psychodynamic account grounded in psychoanalytic theory—the missing layer of psychological depth that links structure to lived experience.

The Frommian framework thus functions not as a replacement for these established traditions but as a means of formulating testable hypotheses about digital workers’ psychological distress: hypotheses that are theoretically anchored in a structural analysis of platform capitalism and neoliberal governmentality, while remaining methodologically operational within the quantitative, mixed-methods, and qualitative designs familiar to work psychology. Its bridging function is to make available to empirical psychology a set of historically informed and incisive concepts for capturing the subjectivity predicament of digital labor. This, precisely, constitutes the article’s distinctive contribution.

## 4 Theoretical construction: digital marketing character, psycho-emotional deficitization, and escape from freedom

This chapter constitutes the theoretical core of the entire article. We will sequentially develop three interrelated theoretical propositions: (1) digital labor cultivates a historically specific type of social character—“digital marketing character”; (2) the psychological effect of this character structure is a diffuse state of “psycho-emotional deficitization”; and (3) this deficit experience and the psychodynamic of escape from freedom are intertwined, driving a recurrent cycle of self-commodification and emotional exhaustion.

### 4.1 Digital marketing character: the contemporary transformation of Fromm’s “marketing orientation”

In *Man for Himself*, Fromm proposed the “marketing orientation” character type. “The character orientation which is rooted in the experience of oneself as a commodity and of one’s value as exchange value I call the marketing orientation” (Fromm, 1947, p. 68). In the marketing-oriented character structure, “I am as you desire me”—the individual molds and packages the self-according to market demands, rather than defining the self-based on intrinsic qualities.

This character orientation has undergone a qualitative leap under the conditions of digital labor. While the “market” in the traditional marketing orientation remained a relatively abstract metaphor—one needed to “sell” oneself in the job market and the marriage market, but these markets were not visible in real time—in the digital platform economy, the market becomes an enveloping, real-time, omnipresent presence. Ratings, rankings, follower counts, click-through rates, completion rates—these metrics allow workers to see at any moment the “stock price” of their personality on the market (Gandini, 2019; Kellogg et al., 2020; Wood et al., 2019).

This difference is a qualitative transformation, not merely a quantitative intensification. This article proposes the concept of “digital marketing character” to describe the specific form taken by the marketing orientation under the conditions of digital labor. Compared with Fromm’s classic description, the digital marketing character possesses three qualitative features:

First, the compulsoriness of self-curation. In the traditional marketing orientation, individuals needed to “package” their image for specific occasions such as interviews, sales, or social encounters. Under the digital marketing character, self-curation becomes a lifelong task covering all domains of life. Updates on social media, persona maintenance on work platforms, deliberate guidance of client reviews, and even personal hobbies and leisure—all are absorbed into the scope of self-curation, because they can all be converted into content, datafied, and thrown into the market for value validation. This is an authentic mode of existence demanded by the digital market, distinct from performance or pretense: if you do not curate yourself, you cannot be “seen” by the market; and if you are not seen by the market, your very existence becomes suspect (Moore, 2018; Crary, 2013).

Second, the internalization of self-quantification. Digital labor platforms provide a profusion of real-time updating quantitative indicators. Traditional research treats these indicators as external management tools (Kellogg et al., 2020; Parent-Rocheleau and Parker, 2022). This article argues that the deeper psychological consequence of these indicators is that they gradually become the sole coordinate system for workers’ self-evaluation. When a food-delivery rider feels anxious over a negative review, the anxiety stems not only from income loss, but more profoundly from the instantaneous experience of a drop in “self-worth”; when a self-media creator panics over a decline in views, the panic is not merely traffic anxiety, but an intuitive reaction to the existential question, “Am I still valuable?” Quantitative indicators transform from external evaluations into an internal experiential structure—self-worth becomes measurable by numbers, and must be measured by numbers. As Moore (2018) reveals, this practice of self-quantification places workers under a persistent pressure of self-surveillance.

Third, the experience of self-commodification as “autonomous.” This is the most disquieting feature of the digital marketing character. The traditional presupposition of alienation theory is that alienated labor is coerced and unpleasant, and that workers can perceive that “this is not the real me.” The digital marketing character shatters this presupposition—it enables the act of “turning oneself into a commodity” to be experienced as self-expression and self-realization. When a self-media creator transforms her life into content products, when a digital nomad turns travel into work and scenery into a backdrop—they genuinely experience a real sense of pleasure and meaning in the content they create, in the interaction with followers, in the moment of landing the next project. This “realness” is precisely the deepest mode of operation of the digital marketing character, rendering alienation imperceptible because alienation has donned the cloak of “self-realization.”

These three features of the digital marketing character form a mutually reinforcing closed loop: self-curation continuously produces material available for quantitative evaluation, quantitative evaluation continuously supplies signals of self-worth being confirmed or denied, and these signals in turn drive more vigorous self-commodification—all while self-commodification itself is experienced as “I am being myself.” These are not merely intensifications of the marketing orientation; they are transformations reflecting the qualitatively new conditions of platform-mediated labor.

It must be emphasized that the digital marketing character is not a monolithic singular form. Just as Fromm consistently stressed that social character is a “shared nucleus” within a group, rather than an exact replica in each individual, there are significant individual differences in how digital workers absorb and embody this character structure. Some workers may have almost completely internalized the quantitative evaluation system; others exist in a state of “ambivalent identification”; still a few develop varying degrees of “reflective resistance.” These differences partly derive from individuals’ life histories and accessible resources, and partly stem from the individual potential for what Fromm called a “productive orientation”—the capacity to relate to the world through love and reason, and to realize one’s powers in action (Fromm, 1947). This heterogeneity provides the theoretical possibility for understanding the breaking and repair of the escape-from-freedom cycle.

### 4.2 Psycho-emotional deficitization: the affective consequence of the digital marketing character

The cultivation of the digital marketing character is not value-neutral. This article argues that its core psychological effect is a specific inner experience—we borrow the term from Lindebaum and Langer (2024), calling it “psycho-emotional deficitization,” and systematically conceptualize it within Fromm’s theoretical framework.

Psycho-emotional deficitization refers to the process whereby the individual internalizes an external ideal image of the “sufficiently good worker,” bound to constantly shifting market standards, and continuously measures their current state against this ideal, thereby experiencing a diffuse sense of “never being enough” due to the perpetual gap between them. This feeling constitutes a diffuse emotional undertone in self-experience, distinct from specific reactions to concrete failures.

This concept has the following qualitative characteristics:

The non-saturability of deficit: This differentiates it from “insufficiency.” Insufficiency points to a concrete shortfall with an endpoint: “I haven’t yet met this month’s performance target.” Deficit points to a void that can never be completely filled: “No matter how well I do this month, I will have to prove myself again next month.” This is because when the standard of “sufficiently good” is defined by an ever-changing market, “enough” itself is an infinitely recursive concept—you can never catch up to the market, because the market is in constant motion.

The internalized nature of deficit: The key is the suffix “-ization”: external standards have been absorbed as a constitutive component of self-cognition and emotional experience. A digital worker with objectively excellent performance may still feel, on a deep level, that they are not working hard enough or are not valuable enough. The root of deficitization lies in the fact that “they have taken the platform’s standard as the very measure of self-evaluation,” rather than in “whether they have met the platform’s assessment criteria”—they no longer have any other measure.

The diffuseness of deficit: This sense of deficit is not confined to specific situations. It does not merely appear during work but pervades the individual’s entire lifeworld. When a worker scrolls through successful peers’ cases late into the sleepless night, when they are unable to enjoy a weekend without any “output,” when they half-jokingly call themselves “has-been” in front of friends—deficitization is the same undercurrent in all these contexts.

The productivity of deficit: From the perspective of the social system, the high degree of self-drivenness in psycho-emotional deficitization makes it a highly “efficient” psychological state. Because the individual perpetually feels not good enough, they are continuously motivated to “become better”—produce more content, take more orders, chase higher ratings. The stronger the sense of deficit, the more unceasing the flow of labor energy required by society. This is the deep coupling between the digital marketing character and the logic of digital capital: workers fuel the system with their own suffering, while experiencing that suffering as “I am simply not working hard enough.”

Here again, an overly homogeneous picture must be avoided. The intensity and expression of psycho-emotional deficitization are not uniform across individuals. For those who have more comprehensively internalized the digital marketing character, the sense of deficit may manifest as a continuous low-grade fever of anxiety; for those in a state of ambivalent identification, the sense of deficit is often entangled with the struggle between exhaustion and self-doubt; and among those workers who possess stronger productive resources or can access alternative community support, the sense of deficit may be partially transformed into a drive for innovation and the search for connection. Acknowledging this experiential diversity does not weaken the explanatory power of the main thesis; rather, it provides the necessary theoretical scaffolding for future research.

### 4.3 “The fear of becoming superfluous”: escape from freedom in digital labor

At this point, one crucial question remains unresolved: if psycho-emotional deficitization is a painful experience, What is the psychological drive that keeps workers trapped in this painful cycle? Mainstream psychology might explain it through “economic necessity” or “habit,” but these explanations cannot exhaust the phenomenon—many digital workers remain unable to extricate themselves from this cycle even after their economic needs are met. This article seeks an answer from Fromm’s theory of escape from freedom.

In digital labor, formal autonomy—the freedom to accept or reject gigs at will, to go online or offline anytime, to set one’s own pace—is a real experience, yet this autonomy is simultaneously burdensome. When a worker is thrown into formal freedom but lacks genuine inner strength and external resources to bear this freedom, freedom transforms into anxiety. This article argues that, under the conditions of digital labor, this anxiety crystallizes into a specific fear—the “fear of becoming superfluous.”

The “fear of becoming superfluous” refers to the fact that digital workers, beneath the veneer of “autonomy,” deeply experience the fear that they could at any moment be downgraded by the algorithm, eliminated by the market, or drowned in the flood of data. “Superfluous” here has an existential connotation: it is not just about being dismissed by a particular platform, but, in a more fundamental sense, about no longer being “needed” by the world.

This fear has specific structural sources in digital labor. Boundless competition forces workers to face a theoretically infinite pool of global laborers (Wood et al., 2019); algorithmic opacity makes evaluation criteria both palpably present and frustratingly elusive (Kellogg et al., 2020); atomized labor deprives workers of collective identity and the possibility of solidarity (Gandini, 2019). Lang et al.’s (2023) research provides indirect support: perceived algorithmic control significantly triggers burnout experiences among gig workers, suggesting that algorithmic management profoundly intervenes in workers’ affective and motivational systems.

Consequently, workers develop a specific mode of escaping this fear: integrating themselves even more actively into the platform’s logic—updating more diligently, tracking data more closely, self-commodifying more thoroughly. This constitutes a contemporary version of escape from freedom: the individual, by completely abandoning the independent self and being entirely engulfed by market logic, obtains psychological protection from the fear of being “discarded.” Work—ceaseless, self-proliferating work—becomes the refuge from existential anxiety.

### 4.4 An integrated formulation of the theoretical framework

In summary, the theoretical framework of this article can be formulated as a causal dynamic chain with three links:

Link 1 (Structure → Character): The specific labor organization of the digital platform economy—algorithmic management, quantitative evaluation, open competition—cultivates a “digital marketing character.” The core features of this character structure are that workers continuously engage in self-curation, self-quantification, and self-commodification, and experience self-commodification as self-realization.

Link 2 (Character → Experience): The inner experience of the digital marketing character is “psycho-emotional deficitization,” a diffuse, non-saturable, internalized feeling of “never being enough.” This feeling pervades self-experience, constituting an enduring emotional undertone, and is qualitatively different from specific reactions to concrete failures.

Link 3 (Experience → Drive): Psycho-emotional deficitization activates the psychological mechanism of escape from freedom, manifested as the “fear of becoming superfluous”—a deep-seated fear of being eliminated, forgotten, and no longer needed. This fear drives workers to throw themselves even more actively into self-commodification, thereby consolidating and reinforcing the digital marketing character and completing the closure of the entire cycle.

This theoretical framework does not presuppose workers to be rational “need-satisfiers,” nor passive “recipients” simply determined by external pressure. It seeks to reveal a more complex psychological fact: workers suffer in behaviors they themselves choose, while the social-structural roots of this suffering are obscured by the experience of “autonomy,” making the suffering both genuine yet leaving them unable to identify a clear perpetrator—ultimately, workers can only attribute the suffering to the fact that “I am still not working hard enough.” It is necessary to reemphasize that this causal dynamic chain describes a typical psychological pathway reinforced in a statistical sense under a particular labor regime, rather than a fated trajectory that every individual must follow in its entirety. The internal heterogeneity of individual character structures, the degree of preservation of productive potentials, and available resources of social solidarity may all act as buffers or even blockages at various links of the dynamic chain, leaving ample space for future research.

## 5 Empirical operationalization: a methodological roadmap for testing the framework

A legitimate concern is that the theoretical framework remains at the level of speculation without a concrete strategy for empirical validation. In this section, we provide a systematic methodological roadmap specifying how each core concept can be operationalized, what research designs are appropriate, and what testable hypotheses can be derived from the framework.

### 5.1 Operationalizing the core constructs

The digital marketing character comprises three interrelated facets—compulsive self-curation, internalized self-quantification, and self-commodification experienced as autonomy—hypothesized to form a coherent latent construct. Development would proceed through a sequential mixed-methods design. First, qualitative interviews would be conducted to generate an item pool that captures the phenomenological texture of the constructs. This would be followed by expert review and cognitive interviewing. Finally, exploratory and confirmatory factor analyses would be performed in larger samples. Convergent and discriminant validity could be assessed against self-objectification (Noll and Fredrickson, 1998), impression management (Paulhus, 1991), and entrepreneurial self-efficacy scales.

Psycho-emotional deficitization is defined by non-saturability, internalization, and diffuseness, distinguishing it from burnout and negative affectivity. A dedicated Psycho-Emotional Deficitization Scale (PEDS) could be developed with items such as “No matter how well I perform, I feel I should be doing more” (non-saturability); “When I am not being productive, I feel like I am losing my value as a person” (internalization); “Even during my free time, I cannot stop thinking about whether I am doing enough” (diffuseness). Experience-sampling methodology (ESM) could complement trait-level measurement by capturing within-person fluctuations.

The fear of becoming superfluous can be operationalized by adapting existential anxiety scales (Weems et al., 2004) to platform labor, with items such as “I worry that at any moment the platform might discard me.” Escape-from-freedom behaviors—proactive overwork beyond economic necessity—can be measured through self-report, platform activity logs, and time-use diaries.

### 5.2 Explicit hypotheses with observable predictions

The theoretical model translates into the following testable hypotheses:

**Table:** 

Hypothe-sis	Statement
H1	Digital marketing character is positively associated with psycho-emotional deficitization (*p* < 0.05, robust to covariates).
H2	Psycho-emotional deficitization mediates the relationship between digital marketing character and burnout/depression (indirect effect significant, bootstrapped CI excludes zero).
H3	Perceived algorithmic control moderates the character–deficitization relationship, stronger at high control (interaction *p* < 0.05).
H4	The fear of becoming superfluous mediates the effect of deficitization on escape-from-freedom behaviors (indirect effect significant).
H5	Escape-from-freedom behaviors and deficitization form a recursive, self-reinforcing cycle over time (bidirectional cross-lagged paths significant at within-person level in RI-CLPM).
H6	Productive orientation and alternative community validation buffer the deficitization–escape cycle (multigroup SEM shows weaker cycle in high-buffer groups).

Each hypothesis is falsifiable with specific statistical tests: H1 via hierarchical regression, H2 and H4 via bootstrapped mediation, H3 via moderated regression with simple slopes, H5 via random-intercept cross-lagged panel models (Hamaker et al., 2015), and H6 via multigroup SEM.

### 5.3 Research designs

We propose a sequential explanatory mixed-methods program (Creswell and Plano Clark, 2017): Phase 1 (Qualitative)—semi-structured interviews and digital ethnography to ground constructs phenomenologically; Phase 2 (Instrument Development)—scale construction following established procedures (Boateng et al., 2018; DeVellis, 2017); Phase 3 (Quantitative Survey)—large-sample SEM testing of the structural model; Phase 4 (Qualitative Follow-Up)—targeted interviews with extreme or anomalous cases to explore protective configurations.

Specific designs for each construct include: qualitative methods (semi-structured interviews, digital ethnography, narrative analysis, clinical case studies) for phenomenological depth; psychometric scales (CFA, convergent/discriminant validity testing, measurement invariance across platforms and cultures) for reliable measurement; longitudinal studies (three-wave panels, ESM, EMA) to capture recursive cycles and micro-temporal dynamics; SEM for multivariate pathway testing and moderated mediation; and comparative studies across platform types differing in algorithmic management intensity, product nature, and worker-platform attachment. Multi-level modeling can examine how platform-level characteristics moderate individual-level relationships, while qualitative comparative analysis (QCA) can identify configurations producing intense deficitization–escape cycles.

This methodological roadmap demonstrates that the Frommian framework is not only theoretically generative but also empirically tractable, constituting a coherent research program that can be pursued incrementally from qualitative exploration through quantitative testing to comparative multi-platform investigations.

## 6 Discussion

The theoretical contributions of this article can be summarized at three levels.

First, it provides a new set of conceptual tools for psychology to understand the subjectivity predicament in digital labor. Mainstream psychological frameworks are adept at capturing relationships between clearly definable and measurable “variables,” but when it comes to diffuse self-experiences that seem almost unconscious—such as “always feeling not good enough” or “the fear of stopping is more terrifying than continuing to run”—the explanatory power of these frameworks is constrained by their methodological context. The concepts of “digital marketing character” and “psycho-emotional deficitization” proposed in this article are analytical tools constructed within Fromm’s methodological context, possessing a unique epistemological function. They allow us to see that certain forms of psychological distress are not “dysfunctions” at the individual level, but the normal operational results of a specific social character structure. This is fundamentally different from simply renaming existing variables. The methodological roadmap further substantiates this claim by demonstrating that these concepts can be operationalized, scaled, and empirically tested.

Second, it builds a methodological dialogue bridge between mainstream psychology and psychoanalytic social psychology. Psychoanalysis has long been marginalized in the academic landscape of contemporary psychology, partly because its methods are often regarded as lacking operability and testability (O’Donnokoé et al., 2025). This article responds to this concern, but adopts a strategy different from “translating psychoanalysis into standard variables”—it positions the contribution of the Frommian paradigm at the level of the explanatory framework: Fromm’s theory can generate a set of testable propositions, which can be investigated using mainstream psychological research designs, while the source of these propositions is a theoretical tradition with a completely different epistemological foundation. This approach of “paradigm dialogue” answers the call for methodological pluralism in contemporary psychology.

Third, it reintroduces Fromm’s theoretical tradition into the core issues of contemporary psychology. Fromm was a highly influential public intellectual and scholar in the mid-20th century, but has virtually disappeared from contemporary academic psychological literature. This article seeks to demonstrate that Fromm’s theory is not merely an academic legacy confined to textbooks of intellectual history, but a set of analytical tools with unique penetrative power regarding problems of subjectivity in contemporary forms of work (Funk, 1998). In the “autonomy paradox” foregrounded by digital labor, the diagnosis Fromm made 80 years ago—that freedom can become a burden, and that humans may escape freedom by abandoning the self—has acquired renewed empirical relevance. Critically, we have also demonstrated that a return to Fromm does not require ignoring either his limitations or the contributions of other critical traditions; rather, a self-consciously qualified and strategically positioned Frommian framework can serve as a productive bridge between critical theory and empirical psychology.

## 7 Conclusion

Grounded in Fromm’s psychoanalytic social psychology, this article has constructed a theoretical framework to explain the specific forms of psychological distress among contemporary digital workers. We argue that the digital platform economy cultivates a “digital marketing character”: workers continuously engage in self-curation, self-quantification, and self-commodification, and experience this self-commodification as an expression of personal autonomy. This character structure engenders a diffuse “psycho-emotional deficitization”—an inner state of constantly feeling insufficiently good and insufficiently valuable, regardless of objective performance. This deficit experience intertwines with the escape-from-freedom dynamic of the “fear of becoming superfluous,” driving a deep cycle in which overwork and emotional exhaustion repeatedly recur.

Beyond this theoretical core, the article has made three further contributions. It has situated the Frommian framework within the broader landscape of critical traditions—including Foucauldian, psychosocial, psychodynamic, posthumanist, and platform capitalism scholarship—explicitly delimiting its contribution as a strategic bridge rather than a claim to exhaustiveness, and critically examining Fromm’s own limitations regarding universalism, normativity, and historical boundedness. It has argued for the formal resilience of Frommian concepts by distinguishing their formal architecture from their historically specific content, demonstrating that the digital marketing character is a deliberate reformulation, not a projection, of the marketing orientation. And it has provided a systematic methodological roadmap with explicit, testable hypotheses spanning qualitative, psychometric, longitudinal, SEM, mixed-methods, and comparative designs, moving the framework from theoretical speculation into an empirically tractable research program.

On a more general level, this article is an invitation regarding psychological methodology. It seeks to demonstrate that the tradition of psychoanalytic social psychology harbors conceptual resources with unique penetrative power for contemporary social phenomena—resources that can enter academic discourse without sacrificing empirical testability. Social character, alienation, escape from freedom—these concepts point toward a mode of analysis that anchors individual psychology within history and social structure, without dissolving the individual into structure. For understanding digital labor as a contemporary form of work, this mode of analysis offers not only explanations but also a way of reframing psychological distress from “an individual’s illness” to “a structural affliction.”

## Data Availability

The original contributions presented in this study are included in this article/supplementary material, further inquiries can be directed to the corresponding author.
